# A phase 2 study of the first imipridone ONC201, a selective DRD2 antagonist for oncology, administered every three weeks in recurrent glioblastoma

**DOI:** 10.18632/oncotarget.17837

**Published:** 2017-05-12

**Authors:** Isabel Arrillaga-Romany, Andrew S. Chi, Joshua E. Allen, Wolfgang Oster, Patrick Y. Wen, Tracy T. Batchelor

**Affiliations:** ^1^ Stephen E. and Catherine Pappas Center for Neuro-Oncology, Massachusetts General Hospital, Boston, MA, USA; ^2^ Center for Neuro-Oncology, Dana-Farber/Brigham and Women’s Cancer Center, Boston, MA, USA; ^3^ Laura and Isaac Perlmutter Cancer Center, NYU Langone Medical Center, New York, NY, USA; ^4^ Oncoceutics, Philadelphia, PA, USA

**Keywords:** ONC201, Imipridone, GPCR, glioblastoma, glioma

## Abstract

ONC201 is an oral, small molecule selective antagonist of the G protein-coupled receptor DRD2 that causes p53-independent apoptosis in tumor cells via integrated stress response activation and Akt/ERK inactivation. We performed a Phase II study that enrolled 17 patients with recurrent, bevacizumab-naïve, IDH1/2 WT glioblastoma who received 625mg ONC201 every three weeks. Median OS was 41.6 weeks with OS6 of 71% and OS9 of 53%. Seven of 17 patients are alive. PFS6 was 11.8% with two patients remaining on study who continue to receive ONC201 for >12 months. One of these patients had a durable objective response with a secondary glioblastoma possessing a H3.3 K27M mutation, exhibiting regression by 85% in one lesion and 76% in the second lesion. The second patient who continues to receive ONC201 for >12 months remains disease-free after enrolling on this trial following a re-resection. No drug-related SAEs or treatment discontinuation due to toxicity occurred. Plasma PK at 2 hours post-dose was 2.6 ug/mL, serum prolactin induction was observed as a surrogate marker of target engagement, and DRD2 was expressed in all evaluated archival tumor specimens. In summary, ONC201 is well tolerated and may have single agent activity in recurrent glioblastoma patients.

## INTRODUCTION

Glioblastoma is the most frequent adult primary malignant brain tumor. FDA-approved treatment options remain few and the prognosis remains dismal with a median survival of 14.6 months [1] and a 5-year-survival rate of 9.8% [2]. In IDH wild-type tumors, MGMT gene promoter methylation status is the most significant prognostic factor for overall survival with extent of resection, corticosteroid use, and number of lesions also possessing prognostic significance. Standard treatment of glioblastoma includes surgery followed by concomitant radiation and temozolomide followed by maintenance temozolomide for 6 months. Unfortunately, this regimen can be associated with toxicity that is particularly problematic for elderly patients who frequently experience neurotoxicity and Grade 3-4 hematological toxicities [3]. Recurrent glioblastoma has no approved therapy that has been shown to prolong survival in a prospective study. Clearly, new therapies are needed for patients with recurrent glioblastoma.

The TP53 pathway is dysregulated in approximately 85% of glioblastomas [4]. Growth factor signaling is also dysregulated in most glioblastoma tumors, with alterations in EGFR, PIK3CA, PTEN, or FGFRA commonly observed. DRD2 is a G protein-coupled receptor that is overexpressed in glioblastoma and controls growth factor signaling through cross-talk mechanisms involving beta-arrestin and scaffold proteins [5, 6]. DRD2 blockade is sufficient to inactivate growth factor signaling and induce tumor cell death in preclinical models of glioblastoma and other malignancies [6, 7]. Interestingly, epidemiologic studies have shown schizophrenic patients, who inherently have elevated DRD2 signaling, have an increased risk of cancer but that this risk returns to normal with DRD2 antagonists [8, 9].

ONC201 is a selective small molecule antagonist of DRD2 that possesses p53-independent anti-cancer activity with infrequent oral administration [10, 11]. Downstream of target engagement, ONC201 causes activation of the integrated stress response and inactivation of Akt/ERK signaling in tumor cells [11-13]. Preclinical studies have shown that ONC201 induces apoptosis in newly diagnosed and recurrent glioblastoma cells *in vitro*, *ex vivo*, and *in vivo* in a manner that is independent of TP53 mutations, MGMT methylation status, or resistance to temozolomide or radiation [10, 11]. Furthermore, ONC201 penetrates the intact blood-brain barrier in rodents, achieving 5-fold higher concentrations in brain tissue relative to plasma, and has proven effective with a survival benefit in an orthotopic mouse xenograft model of glioblastoma [11].

We conducted a phase II study of ONC201 in patients with bevacizumab-naïve recurrent glioblastoma.

## RESULTS

A total of seventeen patients were enrolled between January and April 2016 prior to study closure for failure to meet criteria for continued accrual. Demographics are provided in Table 1. All patients had progressive/recurrent disease with prior surgery, radiation, and temozolomide. Fifteen patients had measurable disease at baseline. The median time from first diagnosis to enrollment was 17.0 months. The median age at enrollment was 57 years-old (range 22-74). Only 2 patients had methylated MGMT tumors and 7 patients had prior gross total tumor resections. Six patients had >1 baseline lesion and 13 patients used corticosteroids at baseline.

**Table 1:** Patient demographics

Age, median (range)	57 (22-74) y/o
Male : Female	9 : 8
KPS, median (range)	90 (70-100)
Number of baseline lesions, median (range)	1 (1-3)
Prior low grade	4
Prior TMZ/RT	17
*Extent of resection at the latest surgery*	
Subtotal	9
Gross total	7
Unknown	1
Salvage surgery at time of recurrence	6
*MGMT*	
methylated	2
unmethylated	13
unknown	2
Corticosteroid use	13

**Figure 1:**
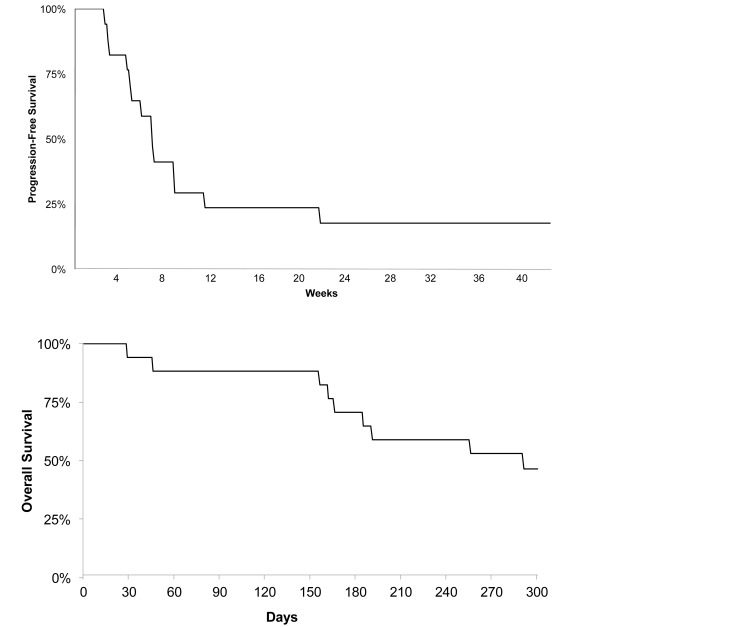
Kaplan–Meier curve for (A) progression-free survival and (B) overall survival

The median number of cycles of ONC201 (21 days) administered was 3 (range 1-18+). Two patients achieved PFS at 6 months and remain on therapy for >11 months, yielding a PFS6 of 11.8% (Figure 1A). Median overall survival was 41.6 weeks, OS6 was 71%, and an OS9 of 53% (Figure 1B). After discontinuation of the trial, 13/15 patients received bevacizumab for some period of time, either as monotherapy or in combination with another treatment.

**Figure 2:**
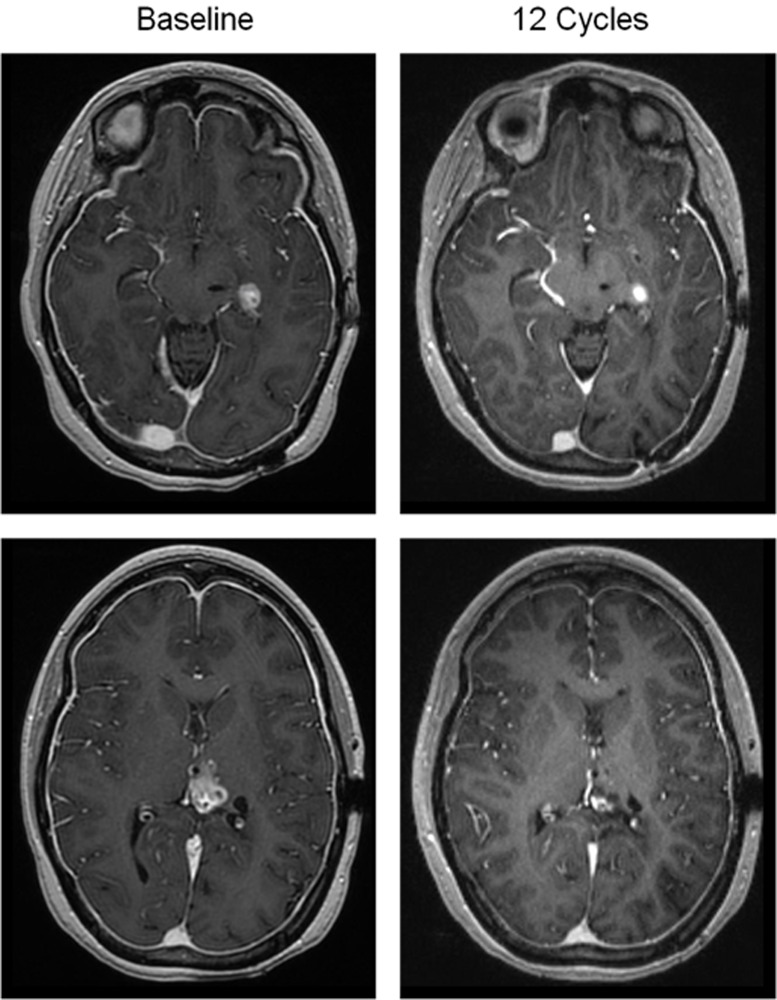
Objective response in a patient with recurrent glioblastoma T1- post contrast brain MRI reveals two enhancing lesions at baseline (left top and bottom panels), which are decreased in size following 12 cycles of therapy (36 weeks; right top and bottom panels).

One 22 year-old female with a recurrent secondary glioblastoma that advanced from a H3.3 K27M mutant Grade III astrocytoma achieved a partial response by RANO occurring after 7 doses, and the response has been sustained for >6 months (Figure 2; Supplemental Figure 1). Two lesions were present at baseline that continuously regressed over time, with one lesion regressing approximately 85% in size compared to baseline after 8.4 months of therapy. The second lesion regressed to approximately 75% of the baseline size after 10.7 months of therapy. The second of these two patients who remain on study drug, a 52-year-old female who initiated ONC201 approximately 7 weeks after salvage surgery, remains disease-free for >11 months.

Only 2 treatment-emergent adverse events occurred on study and there were no treatment discontinuations due to toxicity. One patient experienced one instance of Grade III neutropenia after 5 doses of ONC201 that was transient and did not recur upon re-treatment. The same patient experienced a Grade II allergic reaction after the fifth dose that was managed with anti-histamines, a desensitization protocol, and permitted treatment continuation.

**Figure 3:**
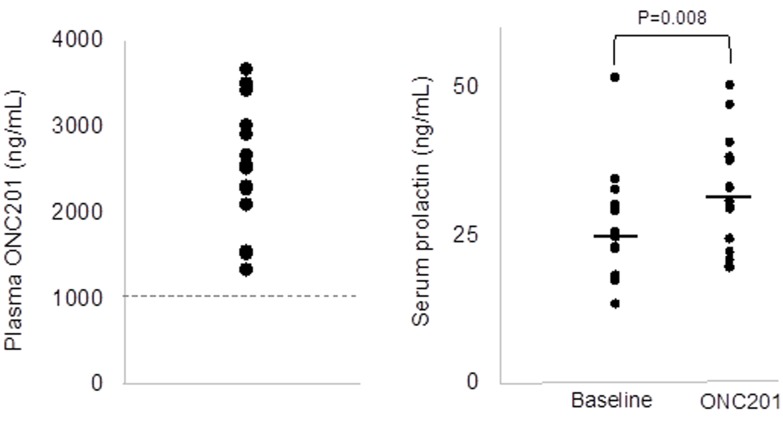
Correlative assays in peripheral blood Left panel shows plasma concentrations of ONC201 at 2 hours following the first dose of ONC201. The dashed line indicates the target therapeutic threshold. Right panel shows serum prolactin levels at baseline and one day following ONC201 treatment.

Analyzing ONC201 plasma concentrations at 2 hours post-dose revealed that every patient achieved drug levels above the target threshold of 1 ug/mL (Figure 3A). As in other clinical studies, induction of prolactin was observed with ONC201 treatment that is classically indicative of DRD2 antagonism in the pituitary gland (Figure 3B). Archival tumor specimens were available for 15/17 patients and all evaluated samples exhibited expression of DRD2 (Supplemental Figure 2).

## DISCUSSION

Although the study did not achieve the primary endpoint, single agent ONC201 showed signs of anti-tumor activity in this pilot study of recurrent glioblastoma patients that was enriched for several poor prognosis features: unmethylated MGMT, age >50, subtotal resections, and baseline corticosteroid use. While progression-free survival did not achieve the primary endpoint in this small cohort, the durable objective response observed and overall survival results are encouraging with a single agent with infrequent dosing.

The extension of survival by a single dose of ONC201 was shown previously in preclinical glioblastoma studies and may be explained by its prolonged pharmacodynamic activity [11]. Cancer stem cells have been shown to express relatively high levels of DRD2 compared to the bulk population [14] and ONC201 effectively depletes cancer stem cells in numerous malignancies [15]. This effect may contribute to the survival prolongation in the patients in this study despite limited exposure to drug.

The durable objective response occurred in the lone patient with a secondary glioblastoma that possessed a H3.3 K27M mutation. Further investigation of the relevance of this mutation to the activity of ONC201 may be warranted, especially given lack of effective treatment options for H3.3 K27M mutant gliomas and the prevalence of this mutation in diffuse intrinsic pontine glioma (DIPG). The kinetics of the response were unusual, exhibiting delayed yet sustained regressions of both lesions that mimics the unusual response kinetics of ONC201 in preclinical models. These slow response kinetics are consistent with those reported in other refractory cancer patients treated in a Phase I study of ONC201 in advanced solid tumors [16]. Furthermore, these response kinetics are similar to responses observed with immune checkpoint inhibitors. The immunomodulatory activity ONC201 has been recently reported, involving increased circulating and intratumoral activated NK cells that may contribute to the antitumor activity of the compound [17, 18].

Another notable observation in this study is the patient who enrolled after salvage surgery and remains disease-free after >11 months of receiving ONC201. Extending disease-free survival is an interesting setting where ONC201 may be well suited given its safety profile and its ability to deplete cancer stem cells. These two observations represent distinct clinical opportunities for ONC201 that will be investigated in dedicated studies.

ONC201 is the first selective DRD2 antagonist under clinical development for oncology. DRD2 has emerging as a novel therapeutic target in glioblastoma and other cancers based on a series of studies that have demonstrated its selective overexpression in malignant tissues and the anticancer effects of its antagonism [6, 7]. The ability of ONC201 to control tumor growth via several signaling pathways downstream of its binding target that is abundantly expressed is a novel therapeutic approach in glioblastoma and other cancers.

While the study was ongoing, a Phase I study has determined that ONC201 is similarly well tolerated at 625mg when administered every week, rather than every three weeks. This more frequent regimen may allow patients with rapidly progressing disease, like glioblastoma, to receive more therapeutic exposure in a short time interval. The efficacy of this weekly regimen is now being evaluated in glioblastoma patients at first recurrence.

Consistent with other clinical studies, ONC201 was well tolerated. The allergic reaction that occurred in one patient in this study is the first reported instance of such an adverse event with ONC201 and was manageable. Importantly, this safety profile was observed with a dosing regimen that achieved or surpassed targeted pharmacokinetic and pharmacodynamic effects. The activity of the drug observed in this study indicates that ONC201 can achieve therapeutic concentrations in the brain, which has been demonstrated in animals and is in concordance with its large volume of distribution observed in Phase I studies.

In conclusion, these results in a pilot cohort of recurrent glioblastoma patients warrants further investigation of ONC201 in gliomas.

## MATERIALS AND METHODS

### Patient population

Eligible patients were ≥ 18 years of age with World Health Organization (2007) Grade IV histologically confirmed diagnosis of glioblastoma who were previously treated with temozolomide and radiotherapy. Patients were required to have unequivocal evidence of progressive disease by RANO and be at least 12 weeks from radiotherapy. Any number of relapses or prior therapies was allowed if Grade >1 adverse events resolved. Prior bevacizumab was not allowed and patients with tumors harboring known IDH1/2 mutations were excluded. Corticosteroid dose were required to be stable or decreasing for at least 5 days prior to the baseline clinical imaging. Concomitant use of potent CYP3A4/5 inducers, which include enzyme inducing antiepileptic drugs, was not allowed during the treatment phase of the study and within 2 weeks prior to starting treatment. Archival tumor tissue was collected, if available.

### Protocol design

ONC201 was administered at 625mg every 21 days as oral capsules that were provided by Oncoceutics. Blood samples were obtained for plasma drug concentration analysis and serum pharmacodynamics. One cycle defined as 21 days. Treatment was continued until tumor progression.

### Patient evaluation

Pretreatment evaluation included a complete medical history and physical and neurologic examinations. Clinical and imaging evaluations were performed every 8 weeks. Imaging was performed using either contrast-enhanced magnetic resonance imaging or computed tomography, maintaining the same modality throughout the study. Response assessments were performed using RANO criteria. Adverse events were graded according to the NCI Common Toxicity Criteria, version 4.0.

### Molecular correlative assays

Cmax was approximated by a blood sample collected in K_2_EDTA at 2-hours post-treatment initiation, which was approximately the Tmax in the first-in-human clinical trial with ONC201. An LC-MS-MS detection method was used as previously described [16]. Archival tumor tissue was retrieved if available. Serum pharmacodynamic samples were drawn at baseline, Cycle 1 Day 2, and on Day 1 of all subsequent cycles. Prolactin levels in the serum were quantified using ELISA (R&D Systems). Immunohistochemistry assessment of DRD2 expression in paraffin-embedded formalin-fixed tumor tissue was performed with an antibody obtained from Santa Cruz (sc-5303) at a dilution of 1:300 for 15 minutes using the automated Leica Bond Rx followed by dehydration and mounting.

### Statistical considerations

The primary endpoint for this study was the determination of the 6-month progression free survival rate (PFS6). Secondary efficacy endpoints included overall survival and objective response. Among recurrent glioblastoma patients, large meta-analyses of clinical trials evaluating a wide array of salvage therapeutics have reported PFS-6 rates of 9-11% [19-21]. This study was designed to detect a PFS-6 of >30% with an accrual of 30 subjects via a two-stage design with appropriate stopping rules for poor efficacy and unexpected toxicity. Enrollment of 30 patients yields 84% power to detect a 20% difference at an alpha level of 0.05 (one-sided). A futility interim analysis was conducted after enrollment of 17 patients using a binary endpoint PFS rate at two months (PFS-2) to determine whether the accrual should be suspended after seventeen patients had accrued.

## SUPPLEMENTARY MATERIALS FIGURES


