# Supplementary material for: A phase 2 study of the first imipridone ONC201, a selective DRD2 antagonist for oncology, administered every three weeks in recurrent glioblastoma
Source: Oncotarget. 2017 May 12;8(45):79298–304. doi: 10.18632/oncotarget.17837 (PMC5668041; doi:10.18632/oncotarget.17837)
Supplement: Supplementary file 1 [file oncotarget-08-79298-s001.pdf]

A phase 2 study of the first imipridone ONC201, a selective DRD2 antagonist for oncology, administered every three weeks in recurrent glioblastoma

Supplementary Material

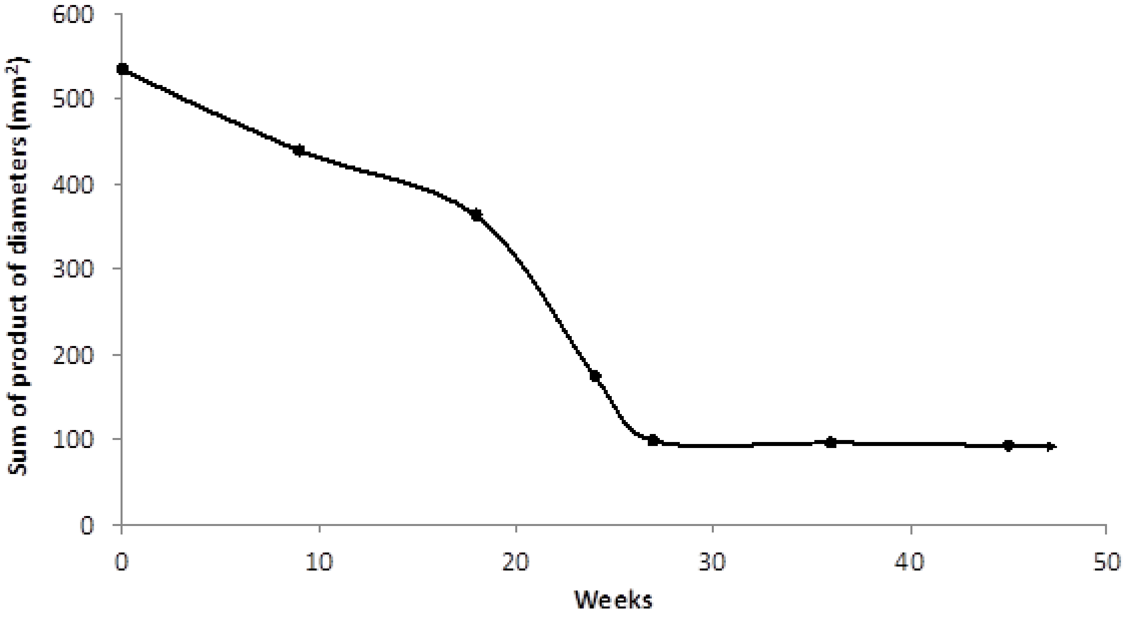

Supplemental Figure 1: Sum of product of diameters of two lesions in patient with objective response by RANO criteria after initiation of ONC201 treatment.

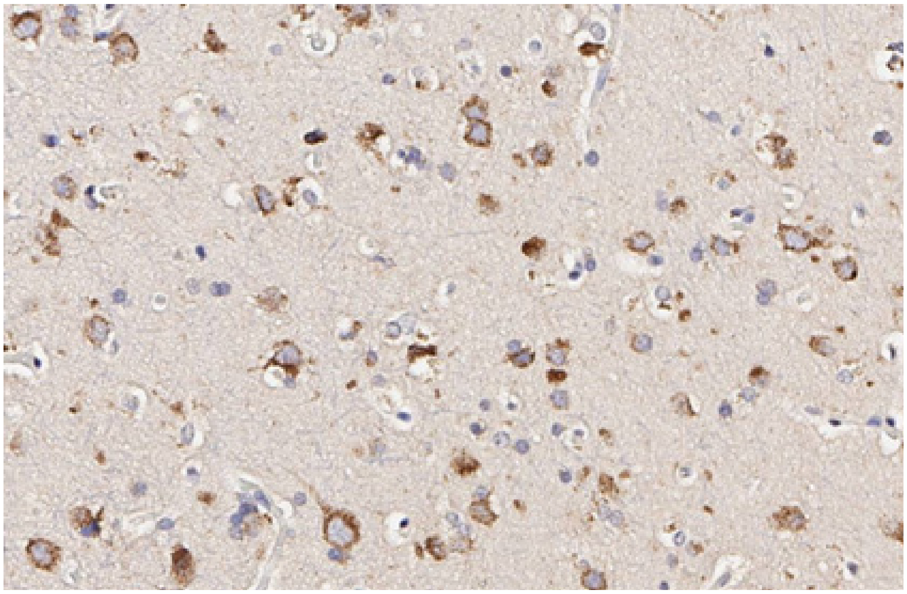

Supplemental Figure 2: DRD2 expression in archival tumor specimen. Exemplary image for DRD2 expression assayed by immunohistochemistry.
